# Brown and beige adipose tissue‐derived metabokine and lipokine inter‐organ signalling in health and disease

**DOI:** 10.1113/EP092008

**Published:** 2024-11-26

**Authors:** Anna Malicka, Aysha Ali, Amanda D. V. MacCannell, Lee D. Roberts

**Affiliations:** ^1^ Leeds Institute of Cardiovascular and Metabolic Medicine, School of Medicine University of Leeds Leeds UK

**Keywords:** adipose tissue, beige adipose tissue, brown adipose tissue, endocrine, inter‐organ signalling, lipokine, metabokine, metabolism

## Abstract

Adipose tissue has an established endocrine function through the secretion of adipokines. However, a role for bioactive metabolites and lipids, termed metabokines and lipokines, is emerging in adipose tissue‐mediated autocrine, paracrine and endocrine signalling and inter‐organ communication. Traditionally seen as passive entities, metabolites are now recognized for their active roles in regulating cellular signalling and local and systemic metabolism. Distinct from white adipose tissue, specific endocrine functions have been attributed to thermogenic brown and beige adipose tissues. Brown and beige adipose tissues have been identified as sources of metabokines and lipokines, which influence diverse metabolic pathways, such as fatty acid β‐oxidation, mitochondrial function and glucose homeostasis, across a range of tissues, including skeletal muscle, adipose tissue and heart. This review explores the intricate signalling mechanisms of brown and beige adipose tissue‐derived metabokines and lipokines, emphasizing their roles in maintaining metabolic homeostasis and their potential dysregulation in metabolic diseases. Furthermore, we discuss the therapeutic potential of targeting these pathways, proposing that precise modulation of metabokine receptors and transporters could offer superior specificity and efficacy in comparison to conventional approaches, such as β‐adrenergic signalling‐stimulated activation of brown adipose tissue thermogenesis. Understanding the complex interactions between adipokines, metabokines and lipokines is essential for developing a systems‐level approach to new interventions for metabolic disorders, underscoring the need for continued research in this rapidly evolving field.

## Abstract (highlights)

- **What is the topic of this review?**
  This review explores the inter‐organ signalling mechanisms of brown and beige adipose tissue‐derived metabokines and lipokines, emphasizing their roles in maintaining metabolic homeostasis and their potential dysregulation in metabolic diseases.
- **What advances does it highlight?**
  We discuss and synergize the existing evidence to explore the therapeutic potential of targeting the metabokine‐mediated pathways to treat cardiometabolic disease, proposing that precise modulation of metabokine receptors and transporters could offer superior specificity and efficacy compared with conventional therapeutic approaches to brown adipose tissue thermogenic activation, such as β‐adrenergic signalling, which have thus far proved unsuccessful.

## 1 INTRODUCTION

Comprehension of the physiological role of adipose tissue was once limited to energy storage and insulation. The discovery of the first adipokine, adipsin, in the white adipose tissue (WAT) of rodents, in 1987, followed by leptin in *ob/ob* mice, in 1994, marked pivotal moments in the recognition of the endocrine role of adipose tissue (Cook et al., 1987; Zhang et al., 1994). These discoveries sparked renewed interest in adipose tissue biology, particularly the discovery of new bioactive signals and molecules produced and released by adipose tissue. It is now recognized that adipose tissue is a dynamic endocrine organ with far‐reaching implications for homeostasis and metabolic health (Scheja & Heeren, 2019).

Adipose tissue is not a single homogeneous tissue type. White adipose tissue acts as an energy reservoir, storing excess energy as triglycerides for later use during periods of increased energy demand or reduced intake. Additionally, WAT provides insulation, aiding in the regulation of body temperature (Trayhurn & Beattie, 2001). Brown adipose tissue (BAT) facilitates thermogenesis through energy expenditure as a means to maintain homeostatic body temperature in response to cold (Horwitz et al., 1969). An emerging phenomenon is the adipogenesis of beige or brown‐in‐white ‘brite’ adipocytes, which are found in WAT depots but have the morphological, functional and metabolic characteristics of brown adipocytes (Wu et al., 2012). The occurrence of beige adipocytes, which are inducible, is thought to occur through two main proposed mechanisms: *de novo* differentiation from progenitor cells (Wu et al., 2012) and transdifferentiation from white adipocytes in response to environmental stimuli (Rosenwald et al., 2013). This process has been termed ‘browning’ and has been observed in both mice and humans. Like BAT, beige adipose tissue (BeAT) can also engage in thermogenesis. Browning is reversible, with beige adipocytes reverting to white adipocytes upon removal of stimuli, a process sometimes referred to as ‘whitening’, highlighting their plasticity (Rosenwald et al., 2013). Since their discovery in adult humans, both BAT and BeAT have been considered promising therapeutic targets for obesity and metabolic disorders owing to their energy‐dissipating influence on systemic energy balance. In humans, who have less BAT and beige adipose tissue compared with rodents, it might be that enhancing *de novo* differentiation in combination with thermogenic stimuli will offer the greatest therapeutic benefit.

Adipose tissue can act as both an endocrine organ and a paracrine organ, producing and releasing various protein and peptide signalling molecules known as adipokines. Adipokines influence both local and systemic physiological functions (Adamczak & Wiecek, 2013), allowing adipose tissue to extend its role beyond energy regulation. Brown and beige adipose tissue secrete specific regulatory adipokine factors known as batokines, which exhibit autocrine and paracrine activities, regulating heat production, hormonal homeostasis, inflammation and metabolism and targeting various organs, such as the heart, liver and skeletal muscle (Villarroya et al., 2017; Yang & Stanford, 2022). Furthermore, BAT indirectly controls processes such as nutritional intake, insulin sensitivity and inflammation (Coelho et al., 2013). Adipocytes not only produce peptide adipokines but also secrete non‐peptide factors, including bioactive metabolites and lipids known as metabokines and lipokines, respectively (Gad et al., 2024; MacCannell & Roberts, 2022; Wang et al., 2023). The recognition of metabokines and lipokines stems from a re‐evaluation of the concept of metabolism, with many metabolites [e.g., amino acid‐derived metabolites, tricarboxylic acid cycle (TCA) intermediates and purines] and lipids now recognized as significant bioactive autocrine, paracrine and endocrine signalling molecules. This group of molecules has been observed to influence systemic energy balance through multi‐organ crosstalk (liver, muscle and pancreas) (Gad et al., 2024; MacCannell & Roberts, 2022).

In this review, we explore BAT and BeAT‐derived metabokines and lipokines, outline the signalling networks through which they mediate tissue crosstalk and discuss their relevance in health and disease as potential therapies or therapeutic targets.

## 2 SHADES OF ADIPOSE TISSUE: BROWN, WHITE AND BEIGE

### 2.1 Anatomical location and distribution

There are notable differences between humans and rodents in the anatomical location, distribution and physiological phenotype of adipose tissue.

In humans, BAT forms during gestation (Velickovic et al., 2014) and plays a crucial role in thermoregulation for newborns, who cannot shiver to generate heat. Initially, BAT is located in the interscapular, cervical, supraclavicular, axillary, paravertebral, mediastinal, periaortic and perirenal regions (Suchacki & Stimson, 2021; Zoico et al., 2019). However, BAT mass and activity decline with age (Pfannenberg et al., 2010). Peripheral depots are the first to lose BAT, with BAT in the interscapular region disappearing soon after birth. Deeper depots, such as those in the periaortic and perirenal regions, decline later in life, and by late adulthood BAT remains localized to the supraclavicular, paravertebral and perirenal regions at reduced levels (Zoico et al., 2019). This age‐related decrease might be a limitation to the utility of BAT as a therapeutic target for cardiometabolic disease.

In rodents, BAT is concentrated predominantly in the interscapular region, unlike the more dispersed distribution in humans (Suchacki & Stimson, 2021). Additionally, rodents retain BAT mass and activity with age, although more recent evidence suggests that ageing mice might exhibit whitening of BAT (Wang et al., 2022).

In both humans and rodents, WAT is divided primarily into two categories: subcutaneous adipose tissue (SAT), located beneath the skin; and visceral adipose tissue (VAT), surrounding internal organs in the body cavity (Mittal, 2019). Expansion of SAT in the lower body to store excess lipids is associated with metabolic health, whereas expansion of VAT and even SAT in the abdomen is linked to metabolic dysregulation, increasing the risk of metabolic disease (Manolopoulos et al., 2010). With age, there is a redistribution of WAT, leading to the loss of SAT and accumulation of VAT (Zoico et al., 2019). Beige adipocytes arise in WAT depots, primarily within SAT rather than VAT depots (Seale et al., 2011). This might be attributed to the genetic make‐up of SAT, which increases susceptibility to browning stimuli (Hildebrand et al., 2018). However, the browning of SAT does decline with age, resulting in reduced levels of BeAT (Zoico et al., 2019). Alongside age, other factors, such as sex, genetics, diet and physical fitness, also play significant roles in driving changes in adipose tissue location, distribution and function. The apparent plastic and responsive nature of adipose tissue to these genetic and environmental pressures undoubtedly plays a key role in susceptibility to multiple metabolic diseases but also offers huge potential for therapies.

### 2.2 Adipogenesis

**FIGURE 1:**
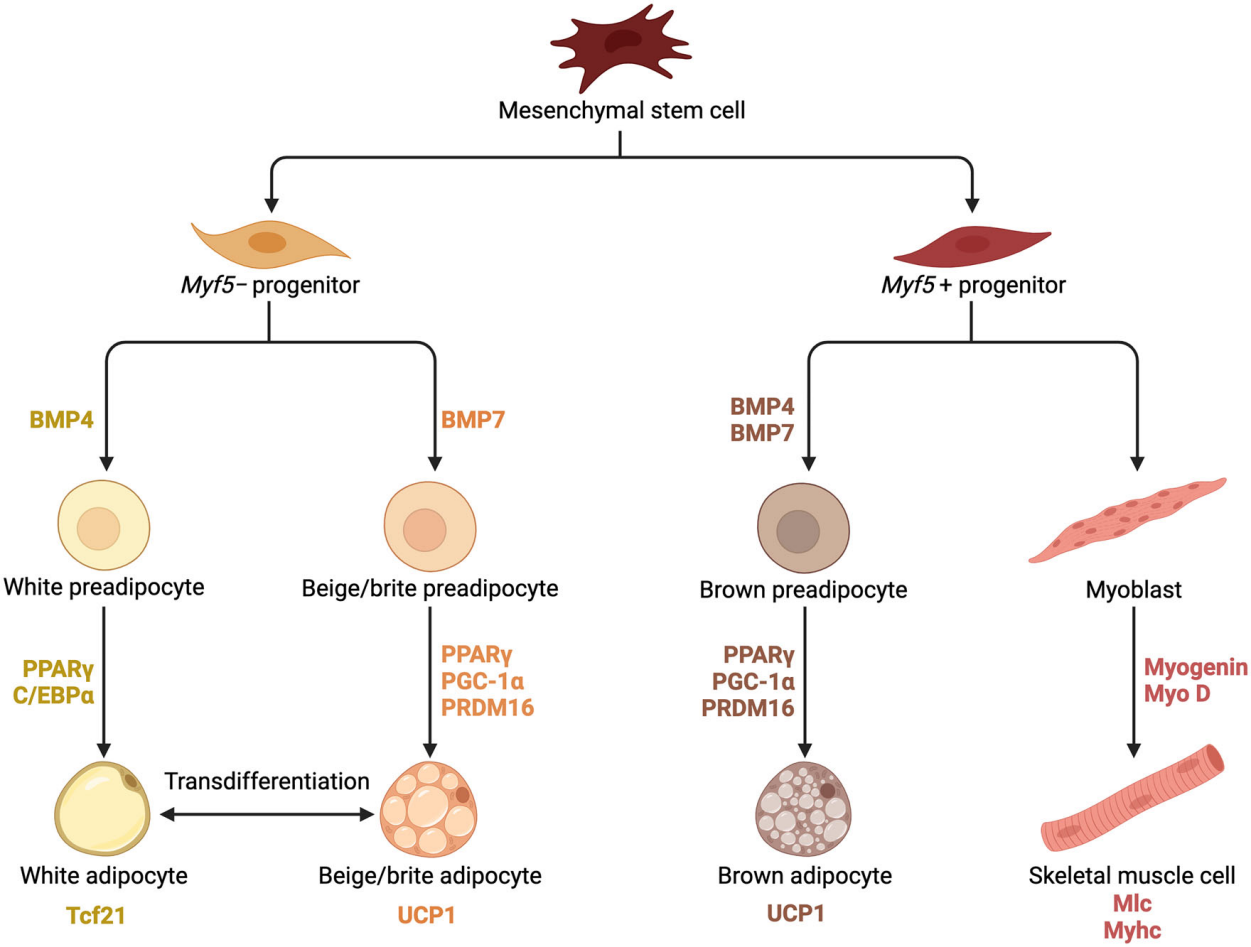
Adipogenesis of white, beige and brown adipocytes from mesenchymal stem cells. Under the influence of bone morphogenic protein 4 (BMP4) and BMP7–small mothers against decapentaplegic (SMAD) signalling, myogenic factor 5‐positive (Myf5^+^) progenitors give rise to brown preadipocytes. Peroxisome proliferator‐activated receptor co‐activator‐1α (PGC‐1α)–peroxisome proliferator activated receptor γ (PPARγ)–PR domain‐containing 16 (PRDM16) signalling then directs their differentiation into uncoupling protein 1 (UCP1)‐expressing brown adipocytes. Decreased PRDM16 upregulates myogenin and myoblast determination protein 1 (MyoD), driving differentiation of myoblasts into skeletal muscle cells expressing myosin light chain (Mlc) and myosin heavy chain (Myhc) (Seale et al., 2008). BMP4–SMAD and subsequent PPARγ–CCAAT/enhancer‐binding protein‐α (C/EBPα) signalling drives development of Myf5^−^ progenitors into white adipocytes expressing transcription factor 21 (Tcf21). Beige adipocytes have been proposed to arise from browning of white adipocytes or BMP7–SMAD and PGC‐1α–PPARγ–PRDM16 signalling [Adapted from ‘Adipocyte lineage’, by BioRender.com (2024)].

Adipocytes differentiate from multi‐potent mesenchymal stem cells, the developmental origin of both adipocytes and myocytes (Pittenger et al., 1999). The development of adipocytes follows distinct lineage pathways: brown adipocytes develop from the myogenic lineage through myogenic factor 5‐positive (Myf5^+^) progenitors, whereas white and beige adipocytes arise from the adipocyte lineage through myogenic factor 5‐negative (Myf5^−^) progenitors (Timmons *et al.*, 2007) (Figure 1). Adipogenesis involves two stages: commitment, during which progenitors differentiate into preadipocytes, primarily under the influence of the transforming growth factor beta (TGF‐β) superfamily; and maturation, during which preadipocytes differentiate into adipocytes, driven by the ligand‐activated nuclear transcription factor peroxisome proliferator‐activated receptor γ (PPARγ).

The TGF‐β superfamily includes two main subfamilies: TGF‐βs and bone morphogenetic proteins (BMPs) (Herpin et al., 2004). Generally, BMP signalling promotes adipocyte differentiation, whereas TGF‐β signalling inhibits this process (Li & Wu, 2020). In brown adipogenesis, BMP4 and BMP7 phosphorylate small mothers against decapentaplegic (SMAD)1/5/8 proteins, which then form a complex with SMAD4 (Li & Wu, 2020). This complex translocates to the nucleus and upregulates PPARγ, which interacts with the cofactors peroxisome proliferator‐activated receptor γ co‐activator‐1α (PGC‐1α) and PR domain containing 16 (PRDM16) to drive the formation of brown adipocytes, thermogenesis, mitochondrial biogenesis and lipid catabolism (Braga et al., 2013). BMP7‐mediated SMAD signalling has also been implicated in beige adipogenesis (Okla et al., 2015). Conversely, BMP4‐mediated SMAD signalling alone, followed by PPARγ and CCAAT/enhancer‐binding protein‐α (C/EBPα) interaction, upregulates genes involved in lipid metabolism, leading to the development of white adipocytes (Linhart et al., 2001) (Figure 1). It is possible that targeting either to inhibit or activate key steps within these pathways might manipulate the adipose phenotype of an individual and prove a valuable therapeutic approach for a range of diseases from obesity to lipodystrophy.

### 2.3 Phenotype and function

Brown adipose tissue is characterized by abundant mitochondria, multilocular lipid droplets, high vascularity and extensive sympathetic innervation, all of which maximize its capacity for thermogenesis. Beige adipose tissue exhibits molecular characteristics similar to BAT, enabling thermogenesis. Conversely, WAT is distinguished by its unilocular lipid droplets and comparatively limited mitochondrial content, blood supply and sympathetic nerve innervation, reflecting its role in energy storage and insulation (Rui, 2017).

**FIGURE 2:**
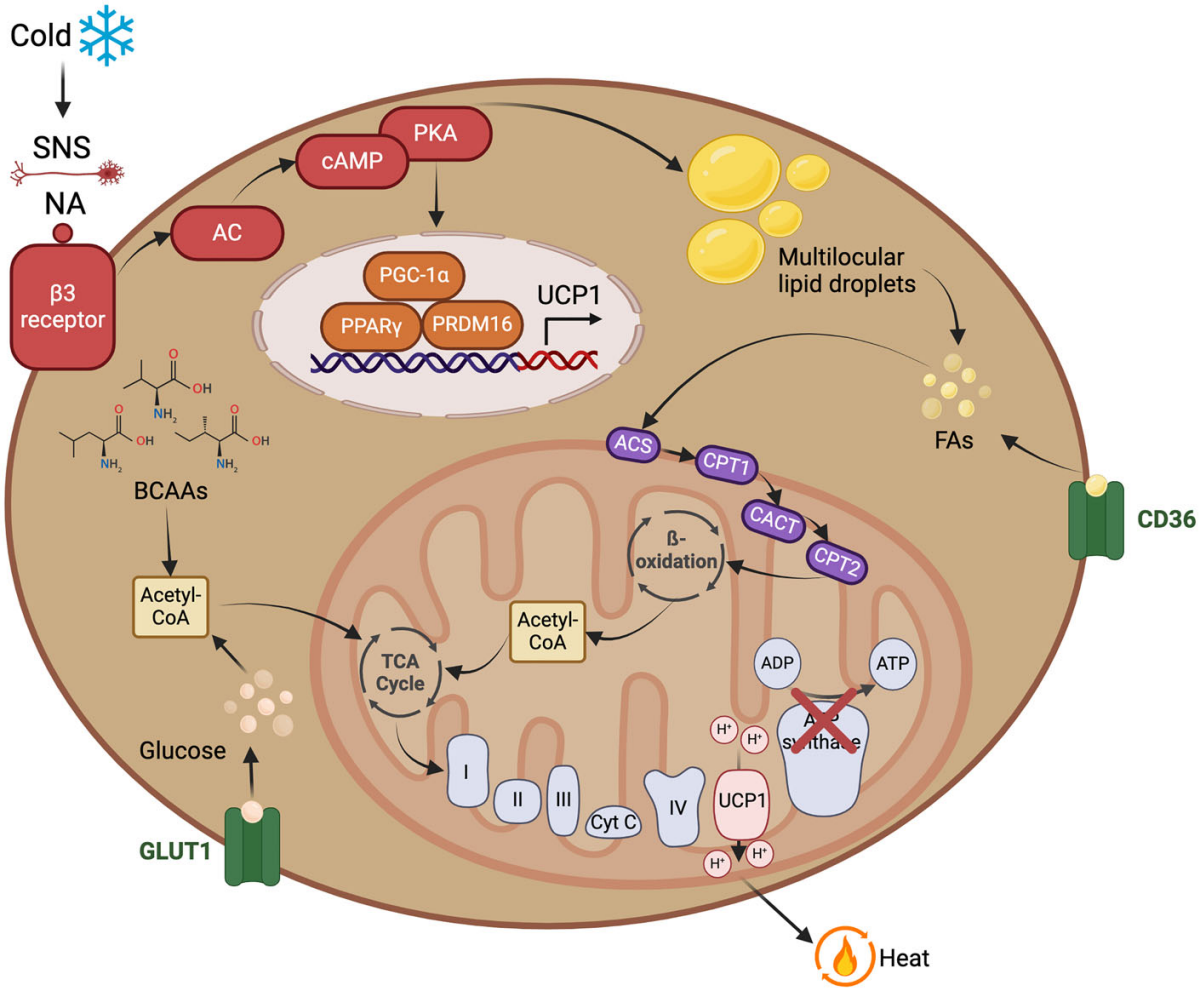
Uncoupling protein 1 (UCP1)‐dependent thermogenesis in brown and beige adipocytes. Cold exposure induces sympathetic stimulation, increasing adenylate cyclase (AC)–cyclic AMP (cAMP)–protein kinase A (PKA) signalling. This promotes peroxisome proliferator‐activated receptor co‐activator‐1α (PGC‐1α)–peroxisome proliferator activated receptor γ(PPARγ)–PR domain‐containing 16 (PRDM16) signalling, increasing mitochondrial biogenesis and UCP1 expression. Long‐chain fatty acids (FAs) derived from lipolysis and cluster of differentiation 36 (CD36)‐mediated uptake enter the mitochondria as acyl‐CoA molecules, via the carnitine shuttle [acyl‐CoA synthetase (ACS), carnitine palmitoyl transferase 1 (CPT1), carnitine‐acylcarnitine translocase (CACT) and CPT2]. Subsequent β‐oxidation of acyl‐CoA produces acetyl‐CoA, which can also arise from metabolism of branched‐chain amino acids (BCAAs) and glucose obtained from glucose transporter protein type 1 (GLUT1) uptake. Acetyl‐CoA enters the tricarboxylic acid (TCA) cycle, generating nicotinamide adenine dinucleotide (NADH) and flavin adenine dinucleotide (FADH_2_) for the electron transport chain (ETC). UCP1 uncouples oxidative phosphorylation, dissipating the proton gradient as heat (Doucette et al., 2023) (created with BioRender.com).

In both humans and rodents, cold exposure is a potent stimulus for inducing non‐shivering thermogenesis in BAT and BeAT (Figure 2). Other factors include exercise, pharmacological and physiological PPARγ ligands, tissue injury and cancer cachexia (Ikeda et al., 2018). In humans and rodents, non‐shivering thermogenesis begins with the sympathetic release of catecholamines, such as noradrenaline (NA), and their subsequent binding to β_3_‐adrenergic receptors on brown and beige adipocytes (Cypess et al., 2015). This initiates a signalling cascade activating adenylate cyclase (AC) production of cyclic adenosine monophosphate (cAMP) and subsequent protein kinase A (PKA) activation. Signalling downstream of PKA upregulates PGC‐1α expression, which enhances mitochondrial biogenesis and complexes with PRDM16 and PPARγ to upregulate uncoupling protein‐1 (UCP1) expression (Cao et al., 2004; Kajimura, 2015; Rui, 2017). Long‐chain fatty acids (FAs) derived from PKA‐mediated lipolysis of neutral lipids (e.g., triglycerides) are transported into the mitochondrial matrix as acyl‐CoA molecules via the carnitine shuttle, where they undergo β‐oxidation, generating acetyl‐CoA (Chen et al., 2022; Kloska et al., 2020; Lee et al., 2015). Acetyl‐CoA is also derived from the metabolism of branched‐chain amino acids (BCAAs) (Carpentier, 2020) and glucose imported from the circulation by insulin‐independent glucose transporter protein type 1 (GLUT1) transporters (Olsen et al., 2014). Acetyl‐CoA enters the TCA cycle, producing NADH and flavin adenine dinucleotide (FADH_2_), which donate electrons to the electron transport chain (ETC) in the inner mitochondrial membrane to drive oxidative phosphorylation. Activated UCP1 uncouples oxidative phosphorylation from ATP synthesis, allowing protons to re‐enter the mitochondrial matrix, dissipating the proton gradient and releasing energy as heat (Rui, 2017) (Figure 2).

Alternatively, UCP1‐independent mechanisms of BAT and BeAT thermogenesis have been identified in mouse models. *N*‐Acyl amino acids (NAAs), formed from the condensation of fatty acids and amino acids catalysed by peptidase M20 domain containing 1 (PM20D1), bind to mitochondria and function as endogenous uncouplers (Long et al., 2016). Thermogenesis can also be induced by the hydrolysis of ATP during creatine futile cycling by creatine kinase (Kazak et al., 2015) and calcium futile cycling by the ATPase (SERCA) pump (Ukropec et al., 2006). The hydrolysis of ATP to ADP and inorganic phosphate (P_i_) in these cycles releases energy, which is dissipated as heat.

### 2.4 Physiological roles in metabolism

Energy imbalance, in a setting of increased intake and decreased expenditure, is the primary driver of mammalian weight gain and adiposity. The storage of excess energy in the form of triglycerides in adipocytes within adipose tissue leads to the accumulation of large hypertrophic adipocytes and excess adiposity (Spalding et al., 2008; Van Baak & Mariman, 2019). Brown and beige adipose tissue‐mediated thermogenesis contributes to energy expenditure and helps to maintain energy balance, which is crucial in body weight regulation. Therefore, a decline in BAT and BeAT mass and activity, as in ageing, can increase the risk of obesity and metabolic disorders.

Brown adipose tissue contributes to cardiometabolic health. The presence and activity of BAT have been proposed as an indicator of metabolically and physiologically healthy individuals (Lidell et al., 2014). Several studies have demonstrated a positive correlation between BAT and improved blood glucose, triglycerides (TGs) and high‐density lipoprotein (HDL) (Becher et al., 2021; Takx et al., 2016), and humans with active BAT have decreased type 2 diabetes (T2D), coronary artery disease, chronic heart failure, hypertension and dyslipidaemia risk (Becher et al., 2021). It has been suggested that BAT activation can prevent the development of hyperlipidaemia and obesity by the increased uptake of TGs into BAT independent of insulin and insulin resistance (Bartelt et al., 2011). However, this claim is countered by others, who suggest that human BAT is not sufficient to counter cardiometabolic diseases (Carpentier & Blondin, 2023). It is likely that any effective human therapy would need to combine an increase in BAT mass alongside any stimulation of thermogenic activity.

## 3 BATOKINES

Adipokines and batokines are crucial regulators of metabolic homeostasis, influencing glucose, lipid and energy metabolism across various tissues. Both adipokines and batokines play essential roles in maintaining metabolic balance and have been the subject of extensive reviews covering their functions (Clemente‐Suárez et al., 2023; Gavaldà‐Navarro et al., 2022; Yang & Stanford, 2022). Given that the primary focus of this review relates to BAT and BeAT, this section provides a brief overview of key protein and peptide batokines and their impacts on metabolic processes.

Myostatin (MSTN) is a member of the TGF‐β protein family and negatively regulates skeletal muscle growth (Chen et al., 2023; McPherron et al., 1997). Additionally, myostatin is inversely related to the thermogenic capacity of BAT and the browning of WAT (Braga et al., 2013; Shan et al., 2013). Using mouse models, Kong et al. (2018), identified that BAT influences skeletal muscle function by secreting myostatin. A deficiency in interferon regulatory factor 4 (IRF4) in BAT, previously identified by Eguchi et al. (2008) as an adipogenesis regulator, leads to elevated myogenic gene expression in BAT and increased myostatin secretion, which, in turn, reduces mitochondrial function and diminishes exercise capacity (Kong et al., 2018). Mice with IRF4 overexpression in BAT exhibited lower serum myostatin levels and enhanced running performance compared with wild‐type mice (Kong et al., 2018). Moreover, exposure to thermoneutral temperatures raises myostatin levels in murine BAT, resulting in decreased exercise capacity (Kong et al., 2018). These observations indicate that myostatin acts as both a negative regulator of BAT and a modulator of skeletal muscle function through its role as a batokine.

Following prolonged cold exposure, BAT expression of fibroblast growth factor 21 (FGF21) in mouse models is elevated compared with the canonical site of expression, the liver (Hondares et al., 2011, 2011; Cuevas‐Ramos et al., 2019). FGF21 regulates the differentiation and thermogenic activation of brown adipocytes, enhancing the expression of UCP1 and promoting the conversion of WAT to BeAT (Chen et al., 2017; Cuevas‐Ramos et al., 2019; Hondares et al., 2011). The influence of FGF21 extends to lipid and glucose metabolism, improving hepatic insulin sensitivity and lipid profiles, with its regulation potentially mediated by miR‐99b secreted from BAT (Falamarzi et al., 2022; Thomou et al., 2017). In the brain, FGF21 reduces sweet‐taste preference and sucrose consumption, whereas its role in the heart suggests involvement in lipid accumulation and oxidative stress (Hill et al., 2019).

Neuregulin 4 (NRG4), a member of the epidermal growth factor (EGF) family, is secreted by BAT (Rosell et al., 2014; Tutunchi et al., 2020). NRG4 is highly expressed in BAT (Wang et al., 2014) and beiging adipocytes (Chen et al., 2024; Wang et al., 2014). NRG4 inhibits lipogenesis and adipogenesis in white adipocytes, promoting lipid uptake and catabolism (Tutunchi et al., 2020; Wang et al., 2014). Secretion of NRG4 by BAT inhibits hepatic *de novo* lipogenesis and reduces hepatic fat accumulation in mice (Blüher, 2019). Recently, a link between NRG4 and the brain has emerged. In mice, BAT‐derived Nrg4 activates receptor tyrosine‐protein kinase epidermal growth factor receptor‐4 (ErbB4) in the hypothalamus, promoting increased release of oxytocin peptides and increased energy expenditure (Zhang et al., 2023).

In mice, elevated levels of phospholipid transfer protein (PLTP) activity have been associated with alterations in lipoprotein metabolism, with the liver serving as a key site for lipoprotein regulation (Yazdanyar & Jiang, 2012). Sponton et al. (2020) have shown that BAT thermogenic activation in mouse models increases circulating levels of PLTP and enhances glucose tolerance, insulin sensitivity and energy expenditure, alongside reductions in circulating cholesterol, phospholipids and sphingolipids. This elevation in PLTP levels is linked to heightened bile acid concentrations in the circulation, consequently promoting glucose uptake into, and thermogenesis within, BAT. These results suggest that PLTP modulates systemic glucose and lipid homeostasis through BAT–liver inter‐organ communication (Sponton et al., 2020). Furthermore, beige adipocytes derived from adipose tissue‐selective PRDM16‐expressing transgenic mice exhibit elevated secretion of PLTP in comparison to white adipocytes isolated from control mice (Sponton et al., 2020), contributing to improved lipoprotein regulation in the liver.

## 4 LIPOKINE AND METABOKINE TISSUE CROSSTALK

**FIGURE 3:**
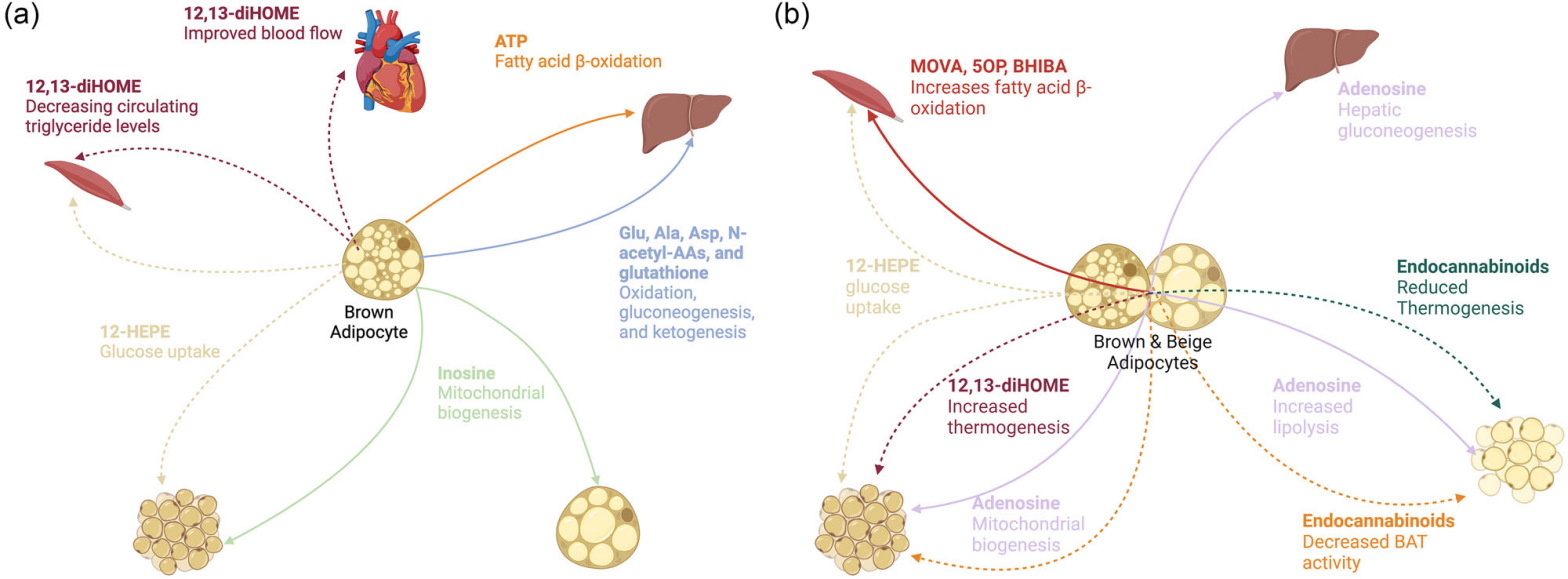
Systemic relationship of metabokines (continuous lines) and lipokines (dotted lines) secreted from brown adipocytes (a) and from beige and brown adipocytes (b), showing the metabokine and lipokine target organs and the physiological and metabolic responses (created with BioRender.com).

Many metabokines and lipokines have been recognized in endocrine axes influencing communication between BAT and BeAT and tissue targets, including the liver, skeletal muscle and pancreas (Li et al., 2020). They are able to act both intracellularly and extracellularly, in some cases simultaneously, providing a range of functions and physiological effects, in addition to an important mechanism for environmental adaptations (Figure 3).

### 4.1 Metabokine‐ and lipokine‐mediated BAT–liver and BeAT–liver crosstalk

The liver plays a crucial role in maintaining systemic glucose and lipid homeostasis throughout fasting and feeding cycles (Jones, 2016).

Purinergic signalling by purine nucleotides and nucleosides, including ATP, uric acid and adenosine, is mediated via four subtypes of purinergic receptors: the inhibitory receptors A1 and A3; and the stimulatory receptors A2A and A2B. Purine signalling specificity is imposed by varying expression levels of these receptor subtypes across tissues in humans and mice (Fredholm et al., 2011; Rines et al., 2015). ATP, the most prevalent intracellular purine, is secreted by brown adipocytes in mice (Gnad et al., 2014; Senthivinayagam et al., 2021). Extracellular ATP is quickly degraded to form adenosine and, subsequently, the more stable purine, inosine (Pfeifer et al., 2024). Inosine circulates through the bloodstream and is taken up by human, mouse and rat hepatocytes via nucleoside transporters (Guinzberg et al., 2006). Inosine is involved in the purine salvage pathway, recycling purines to synthesize ATP, which helps to maintain a steady supply of ATP within rat hepatocytes (Kim et al., 1992). Increased ATP levels resulting from inosine metabolism activate AMP‐activated protein kinase (AMPK). Activated AMPK phosphorylates and inhibits acetyl‐CoA carboxylase (ACC), reducing lipogenesis and mouse hepatic lipid content (Foretz et al., 2018). AMPK activation also enhances carnitine palmitoyl transferase 1 (CPT1) activity, facilitating fatty acid transport into mitochondria for β‐oxidation (Foretz et al., 2018). Inhibition of ACC leads to reduced malonyl‐CoA levels, lowering fatty acid synthesis and lipid accumulation in the liver. By reducing lipogenesis and enhancing fatty acid oxidation, inosine helps to prevent hepatic steatosis, contributing to better hepatic function and metabolic health in mouse models (Cho et al., 2017). Therefore, it could be speculated that BAT‐derived ATP could signal via inosine–AMPK–ACC/CPT1 to mediate hepatic metabolism. Elevated blood adenosine concentrations have also been linked to increased hepatic gluconeogenesis through the inhibition of the suppressive effects of insulin (González‐Benítez et al., 2002; Jain et al., 2021). It is yet to be determined whether this occurs in response to BAT‐derived adenosine during thermogenesis (Jain et al., 2021).

Branched‐chain amino acids (BCAAs) are essential amino acids, meaning that in humans they are not synthesized by the body and must be obtained from the diet, and they include leucine, isoleucine and valine. Brown adipose tissue has emerged as an important site of BCAA oxidation and catabolism, mediating their circulating levels (Verkerke et al., 2024). In mice and humans, catabolism of BCAAs in BAT serves as a key nitrogen source for the synthesis of non‐essential amino acids and their downstream products, including Glu, Ala, Asp, *N*‐acetyl‐AAs and glutathione, many of which are secreted from brown adipocytes (Verkerke et al., 2024). Murine and human liver lacks branched‐chain aminotransferase (BCAT), which is responsible for the reversible transamination of leucine, isoleucine and valine in the BCAA catabolic pathway. The liver depends on the supply of branched‐chain keto acid (BCKA) from other metabolic organs, such as BAT, for oxidation, gluconeogenesis and ketogenesis (Hutson et al., 1992).

### 4.2 Brown adipose tissue–skeletal muscle metabokine and lipokine‐mediated crosstalk

The lipokine 12,13‐dihydroxy‐9Z‐octadecenoic acid (12,13‐diHOME), primarily secreted by BAT, is a regulator of systemic metabolic homeostasis responsible for decreasing circulating triglyceride levels (Stanford et al., 2018). This bioactive lipid is produced by BAT in response to cold exposure in mice and humans (Lynes et al., 2017; Stanford et al., 2018). 12,13‐diHOME is synthesized from linoleic acid by the action of cytochrome P450 enzymes and soluble epoxide hydrolase (sEH) (Halarnkar et al., 1992; Hildreth et al., 2020). Increased production of 12,13‐diHOME in BAT enhances lipolysis and promotes fatty acid uptake into BAT through paracrine signalling. In mice, exercise can also stimulate the endocrine release of 12,13‐diHOME from BAT, increasing fatty acid uptake and mitochondrial fatty acid oxidation in skeletal muscle (Lynes et al., 2017; Rodríguez et al., 2020). 12,13‐diHOME has been correlated with increased mitochondrial respiration in skeletal muscle cells. Studies on differentiated C2C12 myotubes incubated with 12,13‐diHOME reported increased oxygen consumption rate, respiratory capacity and maximal uncoupled respiration (Stanford et al., 2018). C2C12 myotubes incubated with 12,13‐diHOME did not present any changes in glucose uptake efficiency (Stanford et al., 2018). Although emerging evidence links BAT to skeletal muscle adaptation in exercise, as evidenced by the 12,13‐diHOME signal, the physiological importance of this link is more poorly understood and will probably be a focus of future research.

In a study by Whitehead *et al.* (2021) a suite of metabokines released from murine and human thermogenic brown and beige adipocytes via monocarboxylate transporter 1 (MCT1) were identified to signal to skeletal muscle to drive mitochondrial biogenesis, enhance fatty acid oxidation and increase glucose uptake. The metabokines 3‐methyl‐2‐oxovaleric acid (MOVA) and β‐hydroxyisobutyric acid (BHIBA) are released from BAT and BeAT upon catabolism of BCAAs, whereas the metabokine 5‐oxoproline (5OP) is generated in BAT/BeAT through the glutathione synthesis pathway and might provide a link between BAT redox state and systemic adaptation. MOVA and BHIBA require extracellular myofibre receptors to mediate their response in target tissue, whereas 5OP functions through MCT1‐mediated import and intracellular signalling in myocytes. Both 5OP and MOVA were found to signal through a cyclic AMP–PKA–p38 mitogen‐activated protein kinase (p38 MAPK) pathway to control metabolic reprogramming in human primary myocytes (Whitehead et al., 2021; Baker & Rutter, 2023). BHIBA, in contrast, required downstream signalling via mammalian target of rapamycin (mTOR) signalling to mediate effects in skeletal myocytes. Through disparate signalling mechanisms, the metabokines were found to have a synergistic effect on skeletal muscle and myocyte metabolic phenotype in mice when present in combination. The evolutionary pressures that have resulted in BAT and skeletal muscle oxidative metabolism being closely linked through inter‐organ signals remain debated. One potential explanation is a direct physiological link between non‐shivering and shivering thermogenesis in response to cold.

In mice, cold‐induction upregulates the expression and activity of 12‐lipoxygenase (LOX) in BAT, resulting in the production of the lipokine 12‐hydroxyeicosapentaenoic acid (12‐HEPE) (Leiria et al., 2019). This lipokine stimulates glucose uptake in both brown adipocytes and skeletal myotubes through the phosphatidylinositol 3‐kinase–mammalian target of rapamycin—protein kinase B–glucose transporter (PI3K‐mTOR‐Akt‐Glut) signalling pathway (Leiria et al., 2019). The 12‐HEPE‐stimulated increase in GLUT4‐mediated glucose uptake into BAT is accompanied by increased expression of *de novo* lipogenesis genes, generating fatty acids to support the energy requirements of thermogenesis (Gu et al., 2023; Leiria et al., 2019). The increased glucose uptake into BAT and skeletal muscle mediated by 12‐HEPE results in improved glucose tolerance in mouse models of obesity (Leiria et al., 2019; Shaikh et al., 2022). The process of 12‐HEPE‐induced glucose transport might have arisen to support the cellular energy supply required to maintain thermogenesis in BAT and muscle.

### 4.3 Brown/beige adipose tissue metabokine‐ and lipokine‐mediated paracrine and endocrine crosstalk

Brown and white adipose tissue have high levels of purinergic receptor expression, making them a target for BAT‐ and BeAT‐derived adenosine‐mediated purinergic signalling. In both human and mouse BAT, stimulatory A2A receptors are more abundant than inhibitory A1 receptors, whereas in WAT, A1 receptors are more prevalent (Gnad et al., 2014). Consequently, BAT‐ and BeAT‐derived adenosine have opposing effects on these tissues. In BAT, in response to cold exposure, A2A receptor expression is upregulated, and adenosine‐A2A receptor signalling works synergistically with sympathetic stimulation to enhance AC–cAMP–PKA signalling. This promotes mitochondrial biogenesis, *UCP1* expression and lipolysis, increasing thermogenesis and energy expenditure. Conversely, adenosine‐A1 receptor signalling in white adipocytes reduces these metabolic processes by inhibiting AC–cAMP–PKA signalling, leading to a decrease in mitochondrial activity and lipolysis (Gnad et al., 2014). Research indicates that selectively activating A2A receptors, either through A2A agonists or by overexpressing A2A receptors, in WAT can induce browning, leading to increased metabolic activity, which might, potentially, protect against diet‐induced obesity (Gnad et al., 2014).

In mouse models, extracellular inosine, derived from adenosine, activates stimulatory A2A receptors, which increases BAT activity and promotes WAT browning, enhancing thermogenesis and energy expenditure (Niemann et al., 2022). Inosine is released primarily from apoptotic brown adipocytes, potentially as a mechanism to signal for their replacement and influence the metabolic activity of neighbouring adipocytes. Equilibrative nucleoside transporter 1 (ENT1) regulates inosine levels by facilitating its uptake into adipocytes (Ward et al., 2000). This uptake increases intracellular inosine whilst reducing extracellular levels, thereby modulating the thermogenic programme. Impairment or loss of ENT1 function in mice has been shown to reduce inosine uptake into adipocytes, leading to its accumulation in the extracellular space, further enhancing energy expenditure (Niemann et al., 2022). Thus, increasing inosine levels or inhibiting ENT1 can potentially be used to increase thermogenesis and energy expenditure and protect against diet‐induced obesity.

As previously highlighted, cold exposure induces the production and release of the lipokine 12,13‐diHOME from mouse BAT. Interestingly, in cases of BAT deficiency or defect, mouse SAT‐derived BeAT can also secrete 12,13‐diHOME in a compensatory manner (Lynes et al., 2017). This redundancy ensures consistent production of 12,13‐diHOME from either BAT or BeAT even if one tissue is compromised. However, ultimately, secretion of 12,13‐diHOME is higher from BAT compared with WAT (Lynes et al., 2017). 12,13‐diHOME increases energy expenditure of stromal vascular cells isolated from interscapular BAT dissected from mice by driving membrane translocation of fatty acid (FA) transporters, such as CD36 and fatty acid transport protein 1 (FATP1), to enhance FA uptake into brown adipocytes to fuel thermogenesis (Lynes et al., 2017). 12,13‐diHOME also induces browning of WAT depots. Activation of BAT and browning of WAT also contributes to the release of 12,13‐diHOME, creating a virtuous cycle (Lynes et al., 2017).

In mouse models, the BAT and BeAT metabokines MOVA, 5OP and BHIBA can also enhance thermogenic BAT activation and WAT browning via increased FA β‐oxidation, contributing to mitochondrial biogenesis and *UCP1* expression. As in muscle, in BAT and BeAT, MOVA and 5OP signal through the cAMP–PKA–p38 MAPK pathway and BHIBA through the mTOR pathway. Also similar to skeletal muscle, 5OP requires cellular import to induce metabolic gene expression in BAT/BeAT, whereas MOVA and BHIBA function through extracellular signal transduction (Whitehead et al., 2021). In humans, the concentrations of MOVA, BHIBA and 5OP in WAT are positively correlated with the expression of brown adipocyte‐associated genes and inversely with obesity. Obesity is linked to a whitening effect and dysregulated BCAA catabolism in adipose tissue, resulting in decreased biosynthesis and secretion of these metabokines and elevated circulating BCAAs. This disrupts MOVA, 5OP and BHIBA‐mediated inter‐organ signalling, reducing BAT activity and impairing WAT browning. Promisingly, in mouse models of obesity and diabetes, MOVA, 5OP and BHIBA have been shown to reduce adiposity, increase energy expenditure and improve glucose and insulin homeostasis (Whitehead et al., 2021).

Reflecting its effects in skeletal muscle, the lipokine 12‐HEPE also enhances glucose uptake in murine brown adipocytes by activating the PI3K–mTOR–Akt–Glut pathway, which increases the expression of glucose transporters Glut4 and Glut1 on the cell membrane (Leiria et al., 2019). In BAT, glucose uptake contributes to ATP production via glycolysis, offsetting reduced mitochondrial ATP production owing to UCP1‐mediated mitochondrial uncoupling (Vallerand et al., 1990). 12‐HEPE might have therapeutic utility to improve hyperglycaemia and thermogenesis in diabetic patients.

Alternatively, certain lipokines derived from BAT and BeAT, such as the endocannabinoids *N*‐arachidonoylethanolamine [AEA; also known as anandamide (ANA)] and 2‐arachidonoylglycerol (2‐AG), decrease thermogenesis and energy expenditure in mouse models (Krott et al., 2016). These lipid‐based endogenous transmitters become more abundant in BAT and WAT following their respective thermogenic activation and browning via β‐adrenergic signalling. They bind specifically to the cannabinoid type‐1 (CB1) receptor, a G protein‐coupled receptor found on the surface of brown and beige adipocytes. This interaction results in decreased cAMP signalling, serving as a negative feedback mechanism that reverses the activation of BAT and the browning of WAT (Krott et al., 2016). Dysfunction in this system, owing to elevated tissue levels of endocannabinoids and subsequent over‐activation of CB1 receptors in VAT, heightens the risk of fat accumulation and insulin resistance by impairing energy expenditure, whereas CB1 receptor antagonists might improve obesity (Krott et al., 2016).

### 4.4 Brown and beige adipose tissue metabokine‐mediated interactions with the heart

Brown adipose tissue signals to the heart through the lipokine 12,13‐diHOME. Similar to the effect in skeletal muscle, 12,13‐diHOME increases fatty acid uptake within the heart (Pinckard et al., 2021). In addition to increased fatty acid uptake, 12,13‐diHOME improves cardiac blood flow by acting directly on mitochondrial respiration of cardiomyocytes in a nitric oxide synthase type 1‐dependent manner (Pinckard et al., 2021).

## 5 METABOLIC AND THERAPEUTIC IMPLICATIONS

The diverse metabolic pathways and phenotypes regulated by BAT and BeAT‐derived metabokines and lipokines have resulted in significant interest from the perspective of their use as potential therapeutics or as therapeutic targets for metabolic disorders. Their effects on the regulation of systemic energy balance might be a particularly appealing target for diseases such as obesity, insulin resistance and T2D.

### 5.1 Brown and beige adipose tissue activation as a therapeutic for metabolic disorders

Activation of BAT and BeAT has emerged as a promising therapeutic approach for metabolic disorders. Activation of BAT and BeAT promotes energy dissipation through the uncoupling of oxidative phosphorylation, leading to increased calorie expenditure, weight loss, fatty acid and glucose disposal, and improved metabolic parameters. Studies in animal models and humans have shown that enhancing BAT activity through cold exposure, pharmacological agents or genetic manipulation can ameliorate obesity, insulin resistance and dyslipidaemia (Carobbio et al., 2019).

### 5.2 Impact on metabolic disease

Obesity and insulin resistance represent significant public health challenges worldwide, contributing to the development of T2D, cardiovascular disease and other metabolic complications. Through crosstalk with adipose tissue, skeletal muscle and the liver metabokines and lipokines play crucial roles in regulating energy metabolism and insulin sensitivity, and thus might influence the pathogenesis of obesity and insulin resistance.

Alongside the increasing rates of obesity and insulin resistance are incidences of non‐alcoholic fatty liver disease. Humans with increased levels of BAT are at reduced risk of obesity, T2D and non‐alcoholic fatty liver disease (Ahmed et al., 2021; Pinckard & Stanford, 2022). Therefore, the activation of BAT and increased levels of BeAT might be a viable therapeutic option for the treatment of non‐alcoholic fatty liver disease (Srivastava & Veech, 2019). Brown adipose tissue–liver metabokine and lipokine crosstalk might contribute to the beneficial effects of BAT and BeAT activation on hepatic health.

Brown adipose tissue appears to play a distinct protective role in obesity‐associated and obesity‐independent cardiovascular disease, potentially related to anti‐inflammatory effects (Chen et al., 2021). Increased uptake of the glucose analogue ^18^F‐fluorodeoxyglucose (^18^F‐FDG) on positron emission tomography (PET) scans in areas corresponding to supraclavicular fat on computed tomography (CT) images suggests the presence of metabolically active BAT in adult humans (Cohade et al., 2003). Using electronic health records and ^18^F‐FDG PET/CT scans, it was observed that high BAT activity is correlated with lower rates of T2D, dyslipidaemia, coronary artery disease, cerebrovascular disease, congestive heart failure and hypertension, both cross‐sectionally and longitudinally (Raiko et al., 2020). Raiko et al. (2020) found few significant longitudinal correlations between baseline BAT activity and subclinical atherosclerosis over a 5‐year follow‐up in healthy adults; however, negative but borderline significant trends suggest that BAT might be a marker of lower subclinical atherosclerosis levels. These effects are more evident in subjects of normal weight compared with overweight individuals, indicating that BAT activity might have a protective role against subclinical atherosclerosis in healthier populations (Raiko et al., 2020). The effect of BAT activation on cardiovascular system health might be attributable, in part, to direct BAT–cardiovascular system crosstalk, supported by recent discoveries, such as BAT–cardiac crosstalk through the lipokine 12,13‐diHome, discussed above (Pinckard et al., 2021; Tang et al., 2024). Despite its potential benefits, recent research suggests that activated BAT could also accelerate atherosclerosis (Dong et al., 2013; Sui et al., 2019). Therefore, the thermogenic activation of BAT might have a more nuanced role in development of cardiovascular disease. Future therapeutic efforts should focus on defining and translating the specific positive effector mechanisms of BAT on cardiovascular system function whilst minimizing its potential negative effects (Chen et al., 2021).

### 5.3 Pharmacological interventions

A pharmacological strategy for BAT activation has been considered for the treatment of a range of cardiometabolic diseases, including obesity and T2D. By activation of BAT, both the direct effects on energy expenditure mediated by non‐shivering thermogenesis and the indirect effects driven by BAT inter‐organ crosstalk could be harnessed to treat disease.

However, this strategy has resulted in unfavourable off‐target effects, probably attributable to the ubiquity of many of the signalling pathways required for BAT activation (e.g., β‐adrenergic signalling, cAMP–PKA, PPAR). For example, pharmacological agents that activate β_3_‐adrenergic receptors or stimulate the cAMP signalling pathway can promote BAT activation and thermogenesis, leading to improvements in metabolic parameters (Cypess et al., 2015). However, β_3_‐adrenergic agonists have been in development for the treatment of obesity and T2D for >40 years without any being progressed successfully to the clinic for these conditions. This has been complicated, in part, owing to a propensity to induce hypertension and a need for specificity for β_3_‐adrenergic receptors above other subtypes.

Likewise, the pharmacological agents thiazolidinediones, were developed for the treatment of T2D and are thought to improve metabolic health, in part through BAT thermogenic activation and adipose tissue browning (Nedergaard et al., 2005). However, the use of certain thiazolidinediones, such as rosiglitazone, has been associated with increased risk of cardiovascular diseases, including heart failure and myocardial infarction (Nissen & Wolski, 2007).

Given the drawbacks of small molecule strategies aimed at activating BAT thermogenesis, alternative strategies have been explored. Gene editing techniques, such as CRISPR/Cas9, enable precise manipulation of gene expression in adipose tissue, allowing for the modulation of metabokine secretion or adipocyte function. However, direct clinical application is, at present, practically limited. Furthermore, cell‐based therapies involving the transplantation of BAT or BeAT precursors hold potential for restoring metabolic homeostasis in individuals with metabolic disorders (White et al., 2019).

However, given the association of BAT‐derived lipokines and metabokines with improved disease outcomes discussed above, the distinct signalling mechanisms through which they operate and the specificity of target organs mean that metabokines and lipokines are emerging as potential therapeutic interventions.

Metabokines and lipokines secreted by BAT have targeted effects on specific tissues, leading to precise therapeutic outcomes. For instance, adenosine from BAT and BeAT increases mitochondrial biogenesis and lipolysis in BAT, thereby enhancing thermogenesis and energy expenditure, a mechanism that can be exploited to combat obesity (Gnad et al., 2014). We could speculate that a potential indirect mechanism of adenosine secreted from BAT could be to lower fatty acid synthesis and lipid accumulation in the liver through the AMPK–ACC–CPT1 pathway, making it a potential therapeutic for preventing hepatic steatosis (Cho et al., 2017; Gnad et al., 2014; Wang et al., 2015; Woods et al., 2017).

Specific amino acids and their metabolites, such as MOVA and BHIBA, also demonstrate significant therapeutic potential. MOVA enhances fatty acid β‐oxidation in skeletal muscle and BAT through the cAMP–PKA–p38 MAPK pathway, whereas BHIBA does so via the mTOR pathway, making both potential candidates for treating obesity, T2D and metabolic syndrome (Whitehead et al., 2021).

Furthermore, lipokines, such as 12‐HEPE, from BAT enhance GLUT4‐mediated glucose uptake in skeletal muscle and BAT via the PI3K–mTOR–Akt–Glut signalling pathway, improving glucose tolerance and presenting a potential therapeutic target for T2D (Leiria et al., 2019; Shaikh et al., 2022). Additionally, 12,13‐diHOME from BAT reduces circulating TG levels and can be leveraged for treating obesity and metabolic diseases by activating exercise‐ and cold‐responsive pathways (Lynes et al., 2017).

These targeted interventions minimize the broad, systemic effects typically associated with β‐adrenergic or PPAR pathway activators. Although the activation of these broad pathways can indeed boost metabolic rates and improve lipid metabolism, it often comes with systemic side effects owing to their widespread actions (He et al., 2014; Pettersen et al., 2012). For instance, β‐adrenergic agonists can lead to increased heart rate and blood pressure (Yoo et al., 2009), whereas PPAR agonists can cause weight gain and oedema (Guan et al., 2005; Lehrke & Lazar, 2005; Mudaliar et al., 2003). Nevertheless, not all putative metabokines discussed above will be suitable for therapies. Several of the metabokines are ubiquitous, non‐specific and deleterious at higher concentrations. However, by using the more specific metabokines and lipokines, we can achieve precise modulation of metabolic processes, reducing the likelihood of adverse effects and improving overall treatment outcomes.

### 5.4 Supplementation and other strategies

Dietary vitamin supplementation strategies are known to improve metabolic health in a range of conditions (Ford et al., 2003; Melguizo‐Rodríguez et al., 2021; O'Leary & Samman, 2010). Vitamins and micronutrients play essential roles in cellular metabolism, gene expression and signalling pathways that regulate energy homeostasis. Vitamin D deficiency has been associated with obesity, but the specific reasons for this link remain under investigation. Research in mice indicates that inhibiting the vitamin D receptor results in a leaner body type, with an increase in UCP1 levels in both white and brown adipose tissues, along with higher β‐oxidation in WAT (Narvaez et al., 2009). On the contrary, overexpression of the human vitamin D receptor in mouse fat tissue decreases the expression of genes involved in FA transport, thermogenesis and lipolysis, whilst also reducing FA oxidation and lipolysis (Wong et al., 2011). Furthermore, vitamin D influences insulin activity and glucose metabolism in fat cells by boosting insulin‐stimulated AKT phosphorylation, GLUT4 translocation and glucose uptake in 3T3‐L1 adipocytes (Manna & Jain, 2012; Marcotorchino et al., 2012). In mice fed a high‐fat diet, vitamin D supplementation enhances glucose absorption in fat tissue (Manna et al., 2017). However, transcriptome studies in human adipocytes suggest that vitamin D might increase oxidative stress (Sun et al., 2008), potentially impairing insulin signalling pathways. Although some vitamins can exert beneficial effects on metabolic health, others can have adverse effects or lack efficacy in certain populations (Hamishehkar et al., 2016). Moreover, individual tissues can display disparate responses to vitamins, as is the case for vitamin D and metabolic regulation in BAT and WAT. Therefore, a critical overview of vitamin supplementation and other nutritional strategies is essential to identify approaches that optimize metabolic health whilst minimizing potential risks.

Despite the potential limitations of a dietary supplementation strategy, many of the BAT‐derived metabokines identified thus far, including MOVA, BHIBA and 5OP, are physiological, well tolerated, water soluble and orally available. These properties might make them suitable as dietary supplements or amenable to adaption to pharmacological agents.

## 6 CHALLENGES AND FUTURE DIRECTIONS

The study of metabokine signalling presents both exciting opportunities and formidable challenges. As researchers delve deeper into this intricate field, they will encounter various limitations that warrant attention for future advancements.

### 6.1 Current limitations in understanding metabokine signalling

Understanding the complexities of metabokine signalling is a daunting task owing to the intricate network of pathways involved in maintaining metabolite homeostasis. Unlike conventional approaches that target a single gene or pathway, metabokine signalling encompasses multiple interconnected pathways. Disrupting one aspect, such as inhibiting a metabolic enzyme within a biosynthetic pathway, can inadvertently impact numerous physiological processes. This can lead to the accumulation of upstream metabolites and depletion of downstream ones, resulting in unforeseen consequences. Therefore, a nuanced understanding of these pathways is essential to avoid unintended side effects.

The lack of specific inhibitors or activators for metabokines and their pathways hampers the ability to modulate their activity precisely. Traditional pharmacological interventions often lack the specificity required to target individual metabokines (biosynthesis, secretion, receptors) without affecting other related pathways. Developing selective small molecules or gene therapies that can target metabokine signalling pathways precisely remains a significant challenge in the field.

Modulating metabokine receptors or the cellular transporters/exporters of metabokines offers an innovative approach to enhance the specificity and efficacy of treatments for metabolic disorders. This strategy involves targeting the proteins that facilitate the action and movement of metabokines, providing an additional layer of precision in therapeutic interventions. For instance, modulating adenosine receptors in BAT and BeAT can fine‐tune mitochondrial biogenesis and lipolysis, optimizing thermogenesis and energy expenditure to combat obesity without broad systemic effects. Likewise, enhancing the activity of ENT1, the transporter for inosine, could boost thermogenesis selectively in WAT, offering a targeted approach to obesity therapy. These receptor and transporter modulation strategies offer the advantage of precisely controlling the location and intensity of metabokine action, potentially reducing side effects and increasing therapeutic efficacy. By focusing on specific pathways and mechanisms of action, this approach not only complements but also enhances the benefits of using targeted metabokines and lipokines in the treatment of metabolic disorders.

### 6.2 Sex‐specific and age differences

There is a growing recognition of sex‐specific differences in metabokine signalling and its metabolic effects (Chávez‐Guevara et al., 2023). Hormonal differences between human males and females can influence the secretion and response to metabokines, leading to distinct metabolic outcomes (Pauls et al., 2021; Wyskida et al., 2017). Investigating these sex‐specific differences is crucial for developing personalized therapeutic strategies.

Alongside sex differences, ageing is associated with profound changes in metabolism and adipose tissue function. However, the impact of age on metabokine signalling remains largely unexplored (Mancuso & Bouchard, 2019). Understanding how metabokine signalling changes with age and its implications for metabolic health could uncover new therapeutic targets for age‐related metabolic disorders.

### 6.3 Genetic and environmental factors

Genetic predisposition and environmental factors play pivotal roles in shaping metabokine signalling and metabolic outcomes. Variations in the genetic background can influence the expression and function of metabokines (Rhee et al., 2023), and factors such as diet, exercise, environmental contaminants, including pollutants, and stress can modulate their secretion and activity (Lynes et al., 2017). Investigating the interplay between genetic and environmental factors in metabokine signalling could provide valuable insights into individualized approaches for metabolic disease management.

### 6.4 The interaction of adipokines and batokines with metabokine signalling

Adipokines and metabokines share intricate relationships in regulating metabolic homeostasis. Although adipokines originate predominantly from WAT and exert endocrine effects on metabolism, metabokines, including those secreted by BAT and BeAT, have emerged as critical regulators of systemic metabolism. The interplay between adipokines and metabokines is complex and multifaceted. Adipokines such as adiponectin and leptin can modulate the activity of BAT and BeAT and influence the secretion of lipokines such as prostaglandin E_2_ (Lappas et al., 2005). Leptin, known for its role in appetite regulation, also promotes the expression of thermogenic genes in BAT, thereby increasing energy expenditure and inducing the release of lipokines such as 12‐HEPE (Leiria et al., 2019). Another example is resistin, an adipokine that influences hepatic glucose production and has been shown to affect the secretion of BAT‐derived metabolites, such as inosine, which boost thermogenesis (Jain & Jacobson, 2021; Sanches et al., 2023). Understanding the dynamic interactions between adipokines and metabokines is essential for unravelling the complexities of metabolic regulation. Future research efforts should focus on elucidating the crosstalk between these two classes of adipose tissue‐derived signalling molecules and their implications for metabolic health and disease.

## 7 CONCLUSION

In this review, we have explored the complexity of adipose tissue types and the various molecules they release to describe their influence on systemic energy homeostasis and body physiology. Our focus was on BAT‐ and BeAT‐derived metabokines and lipokines, defining these, outlining the signalling networks through which they regulate metabolism and facilitate multi‐organ and tissue crosstalk, and highlighting their relevance in health and disease development. Table 1 summarizes the tissue of origin, target tissue, function and hypothesized therapeutic potential of the metabokine and lipokine signals presented in this review.

**TABLE 1:** Table summarizing the tissue of origin, target tissue, function and hypothesized therapeutic potential of metabokine and lipokine signals

Type	Metabokine/lipokine	Tissue origin	Target tissue	Function	Pathway	Therapeutic potential
Purines	Adenosine triphosphate (ATP)	BAT	Liver (Jain & Jacobson, 2021)	Lowering fatty acid synthesis and lipid accumulation in the liver	AMPK–ACC–CPT1	Prevent hepatic steatosis (Cho et al., 2017)
	Adenosine	BAT and BeAT	Liver	Hepatic gluconeogenesis	cAMP to increase lipolysis (Schimmel & McCarthy, 1984; Zimmermann et al., 2012)	Increase circulating levels of glucose for endurance exercise
		BAT and BeAT	BAT	Mitochondrial biogenesis, *UCP1* expression and lipolysis, increasing thermogenesis and energy expenditure (Gnad et al., 2014)	Adenosine‐A2A increasing AC–cAMP–PKA (Gnad et al., 2014)	Obesity therapies (Gnad et al., 2014)
		BAT and BeAT	WAT	Decrease in mitochondrial activity and increased lipolysis (Gnad et al., 2014)	Adenosine‐A1 decreasing AC–cAMP–PKA (Gnad et al., 2014)	Obesity therapies (Gnad et al., 2014)
	Inosine	BAT	BAT and WAT (browning)	Mitochondrial biogenesis, *UCP1* expression and lipolysis, increasing thermogenesis and energy expenditure (Niemann et al., 2022)	Inosine‐A2A increasing AC–cAMP–PKA (Niemann et al., 2022)	Obesity therapies (Niemann et al., 2022)
Amino acids, branched‐chain amino acids and their metabolites	Glu, Ala, Asp, *N*‐acetyl‐AAs and glutathione (Verkerke et al., 2024)	BAT	Liver	Oxidation, gluconeogenesis and ketogenesis (Hutson et al., 1992)	Catabolism of BCAAs as a nitrogen source for non‐essential amino acids (Verkerke et al., 2024)	Cardioprotective mechanisms against heart failure (Matsuura et al., 2023)
	3‐Methyl‐2‐oxovaleric acid (MOVA)	BAT and BeAT	Skeletal muscle and BAT	Increases fatty acid β‐oxidation (Whitehead *et al.*, 2021)	cAMP–PKA–p38 MAPK; redox stress (Whitehead *et al.*, 2021)	Obesity, T2D and the metabolic syndrome (Whitehead *et al.*, 2021)
	5‐Oxoproline (5OP)	BAT and BeAT	Skeletal Muscle and BAT	Increases fatty acid β‐oxidation (Whitehead *et al.*, 2021)	cAMP–PKA–p38 MAPK; extracellular receptors (Whitehead *et al.*, 2021)	Obesity, T2D and the metabolic syndrome (Whitehead *et al.*, 2021)
	β‐Hydroxyisobutyric acid (BHIBA)	BAT and BeAT	Skeletal muscle and BAT (Whitehead *et al.*, 2021)	Increases fatty acid β‐oxidation (Jang et al., 2016; Whitehead *et al.*, 2021)	Mammalian target of rapamycin (mTOR) (Whitehead *et al.*, 2021)	Obesity, T2D and the metabolic syndrome (Whitehead *et al.*, 2021)
Lipokine	12‐Hydroxyeicosapentaenoic acid (12‐HEPE)	BAT	Skeletal muscle and BAT	GLUT4‐ and GLUT1‐mediated glucose uptake for ATP production and lipogenesis (Vallerand et al., 1990)	PI3K–mTOR–Akt–Glut) signalling pathway (Leiria et al., 2019)	Improved glucose tolerance and T2D (Leiria et al., 2019; Shaikh et al., 2022)
	Endocannabinoids: *N*‐arachidonoylethanolamine (AEA) and 2‐arachidonoylglycerol (2‐AG)	BAT and BeAT	BAT and WAT	Decreased BAT activity and WAT browning, reducing thermogenesis and energy expenditure (Krott et al., 2016)	Endocannabinoid‐cannabinoid type‐1 (CB1) (Krott et al., 2016)	Obesity therapies (Krott et al., 2016).
	12,13‐Dihydroxy‐9Z‐octadecenoic acid (12,13‐diHOME)	BAT	Skeletal muscle	Decreasing circulating triglyceride levels (Stanford et al., 2018)	Exercise and cold activation (Lynes et al., 2017; Stanford et al., 2018)	Obesity and metabolic diseases (Macêdo et al., 2022)
			Heart	Improves cardiac blood flow	Acts on nitric oxide synthase type 1	Heart failure
		BAT and BeAT	BAT	Enhanced FA uptake to fuel thermogenesis (Lynes et al., 2017)	Translocation of CD36 and fatty acid transport protein 1 (FATP1) (Lynes et al., 2017)	Obesity (Lynes et al., 2017)

Abbreviations: ACC, Acetyl‐CoA carboxylase; ATP, Adenosine Triphosphate; AC, Adenylate cyclase; Ala, Alanine; AMPK, AMP‐activated Protein Kinase; 2‐AG, 2‐arachidonoylglycerol; Asp, Aspartic acid; BeAT, Beige Adipose Tissue; BHIBA, β‐hydroxyisobutyric acid; BCAA, Branched‐chain amino acid; BAT, Brown Adipose Tissue; CPT1, Carnitine palmitoyltransferase 1; cAMP, Cyclic adenosine monophosphate; 12,13‐diHOME, 12,13‐dihydroxy‐9Z‐octadecenoic acid; CB1, Endocanniboid‐cannabinoid type‐1; Glut, Glucose transporter type protein; Glu, Glutamic acid; 12‐HEPE, 12‐Hydroxyeicosapentaenoic acid; mTOR, Mammalian target of rapamycin; MOVA, 3‐methyl‐2‐oxovaleric acid; N‐acetyl‐AAs, N‐acetyl‐amino acids; AEA, N‐arachidonoylethanolamine; NOS1, Nitric oxide synthase 1; PI3K, Phosphoinositide 3‐kinase; p38 MAPK, p38 mitogen‐activated protein kinase; PKA, Protein kinase A; T2DM, Type 2 diabetes mellitus; UCP1, Uncoupling protein 1; WAT, White adipose tissue.

Importantly, we have described the clinical relevance of this topic and the potential therapeutic implications for prevalent metabolic diseases, such as obesity, insulin resistance, T2D and cardiovascular disease. However, this field of research is particularly challenging owing to the highly dynamic nature of metabolic signalling, lack of specific pharmacological inhibitors or activators, and significant influence of genetic and environmental factors. Further research to gain a deeper understanding of functions and mechanisms related to adipose tissue, the browning phenomena, and the metabokine and lipokine signals and their pathways affecting multi‐organ crosstalk might lead to new therapeutic advancements.

## AUTHOR CONTRIBUTIONS

All authors reviewed the literature, wrote the manuscript, contributed to the intellectual content and approved the final manuscript.

## CONFLICT OF INTEREST

The authors declare no conflicts of interest.
